# Supplementary material for: Patterns of Intron Gain and Loss in Fungi
Source: PLoS Biol. 2004 Nov 30;2(12):e422. doi: 10.1371/journal.pbio.0020422 (PMC532390; doi:10.1371/journal.pbio.0020422)
Supplement: Table S1 — Also available at http://genes.mit.edu/NielsenEtAl/. (4.3 MB ZIP). [file pbio.0020422.st001.zip › NielsenEtAl/html/896.html]

AN9108.1.NCU01402.1.MG00755.1.FG09040.1


```
 CLUSTAL W (1.82) Multiple Sequence Alignments - Introns Inserted


Sequence 1: AN9108.1	472 aa
Sequence 2: FG09040.1	495 aa
Sequence 3: NCU01402.1	516 aa
Sequence 4: MG00755.1	497 aa
Alignment Length: 550 aa
Number Identitical Residues: 179 aa
Alignment Score (without introns) 9130


MG00755.1 	MGS--LEGLAPAP--DFFVLD-DAKPEDKSLQAFMVSKSRGFLPRMDPVVTLPSEFDPLE
NCU01402.1	MGSQGKDTATYHD--DFPILH-DSRPDDVSIHAFMVSTTRGFLPRMDPIVTLPAEFDPLE
FG09040.1 	MAAANTL---------FPYLRKDPSQLPEGEDPFTITTRTGYLPFSLPLTKLPSQFDPVS
AN9108.1  	MSPATVISSSPTTLESFPHIHDDPSTIPHSLDPFTITTSTGFLPYLTSPTTLPDAFKPLM
          	*..      :    ..*  : .*.     . ..* ::.  *:**   . ..**  *.*: 

MG00755.1 	SILNRMPVKTLSGEPGLLADGR--LGDVVKA-ELPDLTEAMDKY------KDDLPLMNAL
NCU01402.1	SILQRMPVTTASGEPGLLAQGKGQLGEAVDK-ELPDLTEFVDKY------RHDLPLMNAL
FG09040.1 	DLLNDIPIQKLDGTPGLLATFE--LGPLIDNGGLPDLTSEIDNLVVPGTDKKDMAAITAA
AN9108.1  	SLLDRLPVVKHDGSPGLLATYE--LGPAVEQ-ELPDLTDEVDKLVTADG-KLDLYTIMAV
          	.:*: :*: . .* *****  .  **  :.   *****. :*:   ..  : *:  : * 

MG00755.1 	YRDYSFLASAYLLEPCHQRFIKGEE--YGLGRDVLPANIARPIARCAEL2TGFKPFMEYA
NCU01402.1	YRDYSFLASAYLLEPCHERFVIGEE--YGLGRQVLPANVARPIAKCAEI2CEFKPFMEYA
FG09040.1 	FRDYSFIASSYLLEPCWEHYSKSDDGGYGLGRQTLPKCIAGPLVKCAEI2LDIPAFMSYA
AN9108.1  	FRDYSFLASSYLLEPCWKNWRTNPENGYGLGRDVLPKAVARPLYRCGQI2LDIPPFMSYA
          	:*****:**:****** :.:  . :..*****:.**  :* *: :*.::   : .**.**

MG00755.1 	GSYALYNYKLVEPAKG-LEYD---NLRLIRAFEHGLDPLSSEAGFVLVHID1MVRNSGPL
NCU01402.1	GSYALFNYRLVDPSLG-FSFSEPSNVRLIRAFEHGLDPTSSEAGFVLVHID~MVRHSGPL
FG09040.1 	ASYALYNYWLVHPEQGHDDYD---NIRLVRAFEKGLDAKSSEAGFILTHIH~MVAQTGGL
AN9108.1  	ASYALFNYTLDDPSKG-LVYS---NLRLVRAFERGLDPRSSEAGFILTHID~MVKDTPGL
          	.****:** * .*  *   :.   *:**:****:***. ******:*.**. ** .:  *

MG00755.1 	VTGVVEALDACKVAAPGTSRER--------REKVNSG~LAKVLEAMKKINEVMDTMWQKS
NCU01402.1	VAGAVASLDACRRSTLSSSSTHPEMTELSARQSLNVS~LSQMLAALKKINAMMETMWKRS
FG09040.1 	IEGAVDLLTAAEKTDSATKIAE-----------AKAS~LELILNAMQIIETNMEGMWSQS
AN9108.1  	IS----------------------------------A1FLFVMDEYGAHRWRSD-MWANS
          	:                                   . :  ::      .   : ** .*

MG00755.1 	KPRSYTSFRTFIFGITSQSMFPNGVEYEGVS--NGERWDFRGESGANDSI~I--PTCDLL
NCU01402.1	KPDDYTSFRTFIFGITKQSMFPNGVVYEGLPDLNGRPLSFRGESGANDSM~I--PLMDNL
FG09040.1 	LPKDYMTYRTFIYGITKQSMFPDGVVYEGQY--DDKPQFFRGESGANDAI~I--PLLDHI
AN9108.1  	KPSDYLSFRVFIFGITSQSMFPNGVIYDGVE--DNKPLYFRGESGANDSI0IQIPLLDHL
          	 * .* ::*.**:***.*****:** *:*    :..   *********:: *. *  * :

MG00755.1 	LSLPLPTTPLTEILKDFRQYRPSNHRQWLEWVKNSSASYGLKQFALGLDKAD-------D
NCU01402.1	LQIPMPDTPLTKILDDFRSYRPSNHRAFLEWVKRGSEEVGLRRWALALGDSGRGSNDTMT
FG09040.1 	CEVPMPKNPLTDILIEFREYRPKPHRAFLKYVRETAAEVGVRDFLTKSGDLG--------
AN9108.1  	LQIPMPQTPLTEILHEFRSYRPLPHRTFLAHIRNKAEELDVRDFAVEDTET---------
          	 .:*:* .***.** :**.***  ** :*  ::. : . .:: :     .          

MG00755.1 	DNDEAALIQSRALWIQCLDRIREFRWRHW2CFTREFILKQTSHPTATGGSPIVT~WLPNQ
NCU01402.1	EEERAEVRHSRSLWIQVLNQVRDFRWRHW~CFAREYILKQTSHPTATGGSPIVT~WLPNQ
FG09040.1 	---------LAVLYLRLLDHIRSFRWRHW~MFTREYIIKHTLHPTATGGSPIIT~WLPNQ
AN9108.1  	----------AILFLRTLNHVRSFRWRHW~LFAREYIIRRTPHPTATGGSPIVT0WLPNQ
          	            *::: *:::*.******  *:**:*:::* **********:* *****

MG00755.1 	LTAVLDEMANVY------EISTKNGEK-LGEVVD-------DIMDLATRQRDTLAKEVAK
NCU01402.1	LQAVMGHMMGLY------ERMGGDEAGGLGEECK-------DIMELVRRQDETLKKEVRK
FG09040.1 	LGAVMDLMEEVAKGSGLWAVLEEGVWNGGGSLTQEDFILVKKIMDNVVTKKAQLTKEVNK
AN9108.1  	LSAVMDLMISIY-ESVLAPLKAQGTQGYNGSCQK----LVEPMMELVRDQKEKLTKEVEK
          	* **:. *  :   :        .     *.  .      . :*: .  :   * *** *

MG00755.1 	YCEERGVDPAKQGLRV
NCU01402.1	YCEERGVSGA------
FG09040.1 	YCQDRGV---------
AN9108.1  	WCKERGV---------
          	:*::***
```
